# Supplementary material for: Comparative Evaluation of [68Ga]Ga-Fibroblast Activation Protein Inhibitor vs. [18F]FDG as a Novel Radiotracer for Biology-Guided Image Radiotherapy
Source: Cancers (Basel). 2025 Nov 13;17(22):3648. doi: 10.3390/cancers17223648 (PMC12651341; doi:10.3390/cancers17223648)
Supplement: Supplementary file 1 [file cancers-17-03648-s001.zip › cancers-3882463-supplementary.pdf]

Supplementary Information

Figure S1: The chemical structures of [^{68}Ga]Ga-FAPI-04 and [^{18}F]FDG.

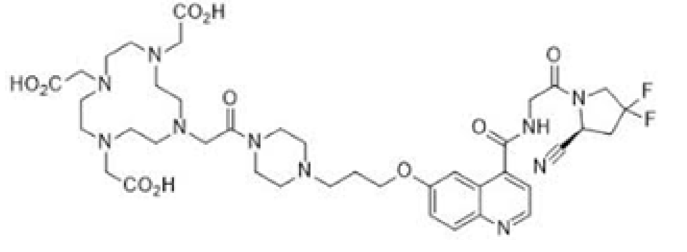	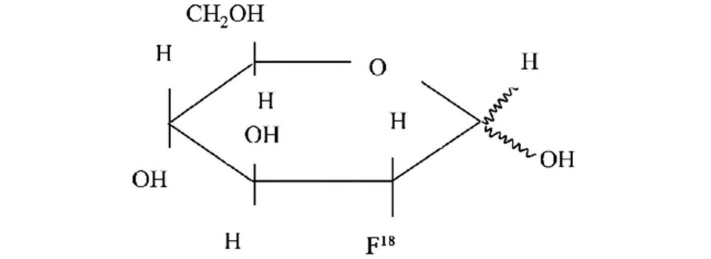
[^{68}Ga]Ga-FAPI-04	[^{18}F]FDG

Table S1: Data table containing patient-level metrics including injected dose, uptake time, and tumor volumes for both tracers.

	Pancreas Patient #	1		2		3		4		5		6		7		8		9		10	
		FAP	FDG	FAP	FDG	FAP	FDG	FAP	FDG	FAP	FDG	FAP	FDG	FAP	FDG	FAP	FDG	FAP	FDG	FAP	FDG
	Injected Activity (mCi)	2.8	7.6	2.7	9.4	3.4	9.2	2.5	9.4	2.7	6.8	3.0	6.6	2.8	6.5	3.3	9.3	3.6	10.0	3.0	7.9
	Elapsed time (mins)	61	62	92	96	67	66	71	61	108	71	29	62	77	51	50	79	46	56	57	57
Physician contours	GTV Volume (cc) drawn by radiation oncologist based on CT and PET	52.3	15.4	40.6	13.1	47.9	13.8	470.5	479.2	17.2	18.3	41.5	31.3	20.1	16.3	17.0	6.3	77.8	28.3	52.3	6.1
	SUVmax	20.9	7.8	12.3	5.2	14.8	11.6	10.2	12.0	12.3	9.8	18.8	4.8	9.7	9.6	10.8	5.9	23.8	10.4	25.5	4.6
	SUVmean	7.1	4.1	4.4	2.9	5.7	5.7	4.4	5.3	6.0	4.7	7.2	2.8	5.4	5.0	5.0	3.9	10.0	5.9	8.0	3.3
Auto-contours	(40% of SUVmax)	8.3	3.1	4.9	2.1	5.9	4.6	4.1	4.8	4.9	3.9	7.5	1.9	3.9	3.9	4.3	2.4	9.5	4.1	10.2	1.8
	GTV_auto (40% of SUVmax)-vol	15.0	12.9	14.9	15.8	21.0	10.4	379.0	263.7	10.0	11.5	18.2	32.3	19.4	12.5	9.7	8.4	38.8	29.3	14.6	9.3
	SUVmax	20.9	7.8	12.3	5.2	14.8	11.6	10.2	12.0	12.3	9.8	18.8	4.8	9.7	9.6	10.8	5.9	23.8	10.4	25.5	4.6
	SUVmean	12.2	4.5	6.8	2.9	8.0	6.5	4.8	6.8	7.8	2.8	10.3	2.9	5.7	5.7	6.5	3.6	14.0	5.9	6.1	3.1
Physician contours	Activity Concentration_Target (Mean AC (kBq/mL) in the volume of GTV receiving 80% of Max kBq)	20.6	25.9	7.9	16.6	14.4	38.3	10.0	43.3	11.5	30.6	33.7	17.0	8.4	32.3	10.7	13.9	26.1	35.3	24.4	14.8
	Normalized Target Signal = (AC_Target - AC_Background)/STD_Background)	22.8	9.2	14.4	7.7	25.0	11.4	12.1	7.3	19.5	11.0	28.4	6.4	4.8	7.2	19.1	5.5	20.6	15.2	36.4	3.5
	Normalized Net Activity Concentration (normalized to injected activity and decay correction)	70.5	19.8	47.4	14.1	51.4	32.8	48.9	36.0	58.0	25.2	67.0	12.5	24.0	23.0	33.7	11.1	78.9	25.6	80.1	8.1

	Liver Patient #	1		2		3		4		5		6		7		8		9		10	
		FAP	FDG	FAP	FDG	FAP	FDG	FAP	FDG	FAP	FDG	FAP	FDG	FAP	FDG	FAP	FDG	FAP	FDG	FAP	FDG
	Injected Activity (mCi)	3.7	9.0	3.5	10.0	3.8	7.8	3.5	11.1	3.7	8.8	3.8	13.4	2.7	8.1	5.6	6.1	9.4	3.4	3.2	9.4
	Elapsed time (mins)	128	59	61	84	55	53	80	91	70	69	63	90	48	69	72	59	72	62	54	63
Physician contours	GTV Volume (cc) drawn by radiation oncologist based on CT and PET	648.3	106.5	1086.3	169.2	139.4	123.5	269	21.7	151.7	156	86.5	12.9	14.1	8.6	475.4	12.5	23	50.4	410.7	301
	SUVmax	15.3	9.7	11.9	16.4	26.8	7.3	12.1	6.2	8.4	12.4	3.9	4.9	9.7	4.6	23.1	8.4	2.3	4.6	14.8	17.6
	SUVmean	4.8	5.35	4.66	6.63	9.94	3.74	2.65	3.17	3.92	5.63	2.1	3.56	4.61	2.51	12.13	4.66	1.51	2.59	2.24	9.14
Auto-contours	(40% of SUVmax)	6.1	3.9	4.8	6.5	10.7	4.6	4.1	2.5	3.3	5.0	1.5	2.0	3.9	1.9	9.2	3.4	0.9	1.8	5.9	7.0
	GTV_auto (40% of SUVmax)-vol	173.9	134.4	505.1	70.0	64.7	24.3	41.9	26.7	97.9	95.8	93.9	16.1	9.2	8.3	324.6	13.8		52.4	24.9	224.3
	SUVmax	15.3	9.7	11.9	16.4	26.8	7.3	14.0	6.2	8.4	12.4	3.9	4.9	9.7	4.6	23.1	8.4		4.6	14.8	17.6
	SUVmean	7.7	5.3	6.2	10.7	14.4	5.3	5.8	3.5	3.2	7.0	2.1	3.4	5.6	2.6	15.1	4.6		2.8	8.2	10.5
Physician contours	Activity Concentration_Target (Mean AC (kBq/mL) in the volume of GTV receiving 80% of Max kBq)	14.2	32.5	11.6	46.7	21.5	21.1	10.3	17.3	11.2	37.1	3.9	23.5	9.6	14.8	49.5	30.4	4.9	5.8	11.3	59.9
	Normalized Target Signal = (AC_Target - AC_Background)/STD_Background	19.9	4.2	14.8	16.6	47.3	5.2	20.7	3.4	12.4	10.6	6.5	3.2	15.6	10.6	19.8	6.0	10.7	1.1	40.8	10.9
	Normalized Net Activity Concentration (normalized to injected activity and decay correction)	83.8	21.4	40.6	47.5	54.6	14.2	46.7	13.6	34.2	33.5	12.9	15.5	28.0	10.7	79.0	19.5	2.1	2.2	39.7	53.4

	Lung Patient #	1		2		3		4		5		6		7		8		9		10	
		FAP	FDG	FAP	FDG	FAP	FDG	FAP	FDG	FAP	FDG	FAP	FDG	FAP	FDG	FAP	FDG	FAP	FDG	FAP	FDG
	Injected Activity (mCi)	2.8	8.6	3.1	9.5	3.6	9.3	2.7	10.3	2.7	7.6	4.4	12.9	3.7	9.6	2.9	8.9	6.2	8.6	5.1	10.1
	Elapsed time (mins)	128	59	61	84	55	53	80	91	70	69	63	90	48	69	72	59	72	62	54	63
Physician contours	GTV Volume (cc) drawn by radiation oncologist based on CT and PET	34.8	38.8	126.0	136.9	17.0	17.3	138.9	106.5	2.8	1.9	2.9	3.8	31.3	23.4	34.8	31.3	51.9	40.4	13.4	9.6
	SUVmax	15.2	20.3	11.6	14.0	8.3	18.5	26.4	9.7	9.9	3.3	7.2	5.7	7.9	8.4	13.1	11.0	21.1	13.0	10.8	9.0
	SUVmean	4.5	6.0	5.9	5.9	2.7	4.5	6.3	2.5	5.3	2.3	3.1	3.6	3.2	4.2	4.7	3.5	6.7	3.4	4.4	3.3
Auto-contours	(40% of SUVmax)	6.1	8.1	4.6	5.6	3.3	7.4	4.1	3.9	4.0	1.3	2.9	2.3	3.2	3.3	5.2	4.4	8.4	5.2	4.3	3.6
	GTV_auto (40% of SUVmax)-vol	10.0	12.1	86.7	67.4	4.9	2.9	102.2	16.1	2.0	4.0	2.0	17.2	15.0	17.4	13.6	5.4	16.1	9.2	6.3	4.8
	SUVmax	15.2	20.3	8.1	14.0	8.3	18.5	26.4	9.7	9.9	3.3	7.2	5.7	7.9	8.4	13.1	11.0	21.1	13.0	10.8	9.0
	SUVmean	9.2	12.1	2.2	4.9	4.6	11.0	7.8	5.1	6.1	2.0	4.6	2.8	4.3	5.0	7.0	6.4	12.3	7.2	7.0	5.6
Physician contours	Activity Concentration_Target (Mean AC (kBq/mL) in the volume of GTV receiving 80% of Max kBq)	6.8	72.6	10.0	39.6	8.8	62.4	15.3	27.8	10.0	11.2	7.0	17.5	9.7	17.4	11.5	43.9	37.1	47.5	14.6	28.2
	Normalized Target Signal = (AC_Target - AC_Background)/STD_Background	53.2	39.4	27.6	20.9	21.1	29.6	50.7	19.7	46.8	7.5	28.1	6.4	22.9	7.9	26.6	16.6	40.2	18.4	23.9	11.1
	Normalized Net Activity Concentration (normalized to injected activity and decay correction)	54.2	69.5	37.9	42.3	27.0	56.2	95.5	30.8	39.7	8.7	25.2	16.1	26.4	14.4	49.1	38.3	74.8	44.1	34.0	25.5

	H&N Patient #	1		2		3		4		5		6		7		8		9		10	
		FAP	FDG	FAP	FDG	FAP	FDG	FAP	FDG	FAP	FDG	FAP	FDG	FAP	FDG	FAP	FDG	FAP	FDG	FAP	FDG
	Injected Activity (mCi)	2.9	8.8	4.0	11.8	3.4	12.3	2.9	9.3	3.4	10.8	2.6	8.1	3.2	10.9	4.4	8.1	2.6	6.9	3.9	12.6
	Elapsed time (mins)	71	77	61	61	69	58	83	61	41	63	61	63	69	64	85	76	65	60	51	69
Physician Contours	GTV Volume (cc) drawn by radiation oncologist based on CT and PET	4.2	2.5	54.3	56.1	136.6	137.2	41.9	49.0	87.9	88.2	25.2	20.4	30.7	13.2	11.4	5.1	28.2	20.1	3.1	2.8
	SUVmax	11.8	15.2	18.5	17.3	13.6	19.9	17.8	13.5	23.6	6.3	16.0	14.1	9.6	16.1	17.4	10.5	23.1	8.4	10.6	4.8
	SUVmean	5.7	8.7	9.0	6.4	2.4	2.3	9.9	7.1	8.3	2.8	7.9	6.1	5.6	8.6	10.8	7.3	9.5	5.0	7.8	3.3
Auto-contours	(40% of SUVmax)	4.7	6.1	7.4	6.9	5.4	8.0	7.1	5.4	9.5	2.5	6.4	5.6	3.8	6.4	7.0	4.2	9.2	3.3	4.2	1.9
	GTV_auto (40% of SUVmax)-vol	3.3	2.8	36.5	19.6	18.2	10.5	43.0	33.6	28.9	56.9	21.7	9.8	25.8	11.5	12.6	11.4	14.2	21.9	4.2	11.2
	SUVmax	11.8	15.2	18.5	17.3	13.6	19.9	17.8	13.5	23.6	6.3	16.0	14.1	9.6	16.1	17.4	10.5	23.1	8.4	10.6	4.8
	SUVmean	7.0	8.6	10.5	9.8	7.8	10.9	10.2	8.6	13.1	3.4	8.9	8.2	6.1	9.2	10.6	6.3	12.6	5.0	5.9	2.6
Physician contours	Activity Concentration_Target (Mean AC (kBq/mL) in the volume of GTV receiving 80% of Max kBq)	8.7	50.5	16.1	58.6	10.7	84.4	12.5	47.9	25.4	21.2	15.5	48.4	9.2	58.0	21.3	34.4	22.8	29.3	10.9	17.9
	Normalized Target Signal = (AC_Target - AC_Background)/STD_Background	9.4	14.4	26.2	9.7	25.5	44.3	9.3	15.1	38.1	12.5	14.6	19.3	15.1	15.2	16.6	7.1	23.2	11.2	7.1	6.0
	Normalized Net Activity Concentration (normalized to injected activity and decay correction)	36.9	31.9	60.9	37.3	52.7	58.9	59.3	31.2	84.9	12.7	58.8	31.5	42.0	35.6	59.4	19.2	80.9	17.2	31.8	8.8

	Cervical Cancer Patient #	1		2		3		4		5		6		7		8		9		10	
		FAP	FDG	FAP	FDG	FAP	FDG	FAP	FDG	FAP	FDG	FAP	FDG	FAP	FDG	FAP	FDG	FAP	FDG	FAP	FDG
	Injected Activity (mCi)	2.5	6.1	3.5	10.5	3.3	8.0	2.6	7.2	2.6	8.4	2.7	8.9	3.3	9.5	2.6	8.5	2.0	9.2	5.2	12.2
	Elapsed time (mins)	57	131	98	95	55	87	68	59	75	67	67	88	70	69	51	70	50	84	66	63
Physician contours	GTV Volume (cc) drawn by radiation oncologist based on CT and PET	30.0	14.7	35.9	28.4	49.9	39.7	19.4	15.6	43.4	30.3	40.8	57.7	21.8	14.5	10.3	3.6	35.0	34.4	49.7	14.6
	SUVmax	22.0	11.4	12.1	9.0	10.7	13.3	19.0	14.9	25.7	15.4	20.9	17.5	19.2	19.4	20.8	6.1	17.2	8.8	27.4	19.3
	SUVmean	9.8	6.6	5.6	5.6	6.2	6.6	9.9	7.9	13.2	7.8	11.2	8.1	10.7	10.2	11.2	4.2	9.9	5.1	11.5	9.5
Auto Contours	(40% of SUVmax)	8.8	4.5	4.8	3.6	4.3	5.3	7.6	6.0	10.3	6.1	8.4	7.0	7.7	7.8	8.3	2.4	6.9	3.5	11.0	7.7
	GTV_auto (40% of SUVmax)-vol	21.0	22.1	41.1	27.6	90.8	41.5	15.0	12.8	32.4	25.4	31.0	31.4	18.9	10.2	6.9	6.1	33.8	37.0	21.7	10.0
	SUVmax	22.0	18.1	12.1	9.0	13.4	13.3	19.0	14.9	25.7	15.4	20.9	17.5	21.5	19.4	20.8	6.1	17.2	9.7	27.4	19.3
	SUVmean	12.3	7.7	6.4	5.7	6.9	6.9	11.0	8.8	15.6	8.8	12.4	10.7	11.7	11.7	13.8	3.7	10.4	5.2	16.1	11.1
Physician contours	Activity Concentration_Target (Mean AC (kBq/mL) in the volume of GTV receiving 80% of Max kBq)	25.1	24.4	7.6	25.1	12.7	39.3	18.1	53.2	19.4	48.2	18.1	57.2	15.9	60.8	23.4	22.9	10.5	39.0	31.6	72.3
	Normalized Target Signal = (AC_Target - AC_Background)/STD_Background)	6.6	3.0	3.5	7.4	1.3	4.7	10.9	3.8	6.0	4.6	2.3	2.7	0.5	6.8	5.5	1.2	2.1	0.8	3.1	15.2
	Normalized Net Activity Concentration (normalized to injected activity and decay correction)	70.5	26.8	34.9	23.5	21.9	35.1	63.7	40.8	80.3	39.5	61.4	53.8	34.5	55.5	85.1	12.7	43.9	27.0	87.4	63.3
